# Antioxidant and Anti-Inflammatory Activity of a New Formulation of Slow-Release Amino Acids in Human Intestinal Caco-2 Cells

**DOI:** 10.3390/antiox14030271

**Published:** 2025-02-26

**Authors:** Carlotta Bollati, Martina Tosi, Lorenza d’Adduzio, Melissa Fanzaga, Alberto Burlina, Gianvincenzo Zuccotti, Carmen Lammi, Elvira Verduci

**Affiliations:** 1 Department of Pharmaceutical Sciences, University of Milan, 20133 Milan, Italy; carlotta.bollati@unimi.it (C.B.); lorenza.dadduzio@unimi.it (L.d.); melissa.fanzaga@unimi.it (M.F.); 2 Department of Pediatrics, Vittore Buzzi Children’s Hospital, University of Milan, 20154 Milan, Italygianvincenzo.zuccotti@unimi.it (G.Z.); 3 Department of Health Sciences, University of Milan, 20142 Milan, Italy; elvira.verduci@unimi.it; 4 Division of Inherited Metabolic Diseases, Department of Women’s and Children’s Health, Reference Centre Expanded Newborn Screening, University Hospital, 35128 Padua, Italy; alberto.burlina@unipd.it; 5 Department of Biomedical and Clinical Science, University of Milan, 20157 Milan, Italy; 6 Metabolic Diseases Unit, Department of Pediatrics, Vittore Buzzi Children’s Hospital, University of Milan, 20154 Milan, Italy

**Keywords:** Caco-2 cells, non-communicable diseases, phenylketonuria, slow-release amino acids, oxidative stress, inflammation

## Abstract

Phenylalanine (Phe)-free protein substitutes (PSs) are used to provide an adequate intake of amino acids (AAs), except Phe, allowing control of blood Phe levels in patients with Phenylketonuria (PKU). Although indicated as a standard dietary treatment for these patients, free AAs mixtures are not absorbed as natural proteins, thus creating an oxidized and inflamed state in the intestine. Nowadays, PSs on the market also include slow-release amino acids (SR-AAs) formulas. The present work aims to investigate the effects of an SR-AAs formula on both oxidative and inflammatory status in human intestinal Caco-2 cells, comparing its mechanism of action with that of a mixture of free AAs. In more detail, oxidative stress and inflammation were induced at the cellular level using H_2_O_2_ and lipopolysaccharides (LPSs), respectively, and both free AAs and SR-AAs PSs were tested to evaluate if they were able to restore a more balanced condition. According to our findings, free AAs aggravate the intestinal oxidative and inflammatory status caused by H_2_O_2_ and LPS in human intestinal Caco-2 cells, which SR-AAs significantly improve. In conclusion, our results offer preclinical novelty on these products’ mechanisms of action, thus improving the dietary management of patients with PKU.

## 1. Introduction

Phenylketonuria (PKU), OMIM: 261600, is the most prevalent inherited metabolic disorder of amino acid metabolism. Mutations of the liver enzyme phenylalanine hydroxylase (PAH) are responsible for the high phenylalanine (Phe) blood levels leading to neurocognitive impairment and psychomotor alterations [1,2,3]. Dietary intervention is the cornerstone for the treatment because dietary Phe restriction allows one to prevent intellectual disability [4]. Along with Phe restriction, by limiting the intake of protein-rich natural foods, the nutritional treatment is based on the use of L-amino acid (L-AAs) protein substitutes (PSs) and Special Low-Protein Foods (SLPFs) [5]. These PSs are also enriched with carbohydrates, lipids, and micronutrients, which are fundamental for the adequate development and growth of the child. The digestion of free L-AAs in PS bypasses the typical digestive process, leading to a distinct absorption pattern compared to whole proteins, with a quicker peak in plasma concentration followed by a rapid decline [6]. Additionally, the faster absorption of free L-AAs results in higher rates of oxidation, protein breakdown, and increased nitrogen excretion in urine [7,8]. Over the years, technological and scientific innovations have significantly expanded the range of PSs commercially available for patients with PKU, aiming to enhance therapeutic adherence to the diet and minimize discomfort associated with the use of L-AAs PSs [9]. Thanks to innovations in food technology, new PSs with the ability to delay the absorption of L-AAs, mimicking physiological absorption of natural intact protein, have been launched on the market, with the aim of improving protein metabolism and minimizing the fluctuations in quantitative plasma amino acid levels [10]. The first prolonged-release PS was developed in 2014 [11], and in 2018 a new prolonged-release PS of L-AAs coated with ethyl cellulose and alginate was launched, with improved taste and smell, in addition to less gastrointestinal symptomatology [12]. In fact, free L-AAs digestion circumvents the digestion phase, resulting in a different absorption profile compared to that of intact proteins, a faster peak, and subsequent decrease in plasma [6]. Moreover, free L-AAs’ faster uptake leads to increased oxidation, protein catabolism, and amount of nitrogen excreted with urine [7]. The aim of slow or prolonged-release PS is to assure a similar absorption profile to natural protein together with a better taste to improve dietary compliance of patients [12]. In healthy adults, slow-release PS lowered the plasma essential AAs (EAAs) peak and sustained the overall bioavailability of EAAs, lowered blood urea nitrogen (BUN) and urea excretion, lowered insulin peak, and showed a less pronounced reduction in blood glucose levels, which could contribute to a normal satiety response [10]. In children with PKU, only one prospective observational study, based on replacing 20–30% of the standard PS intake with prolonged-release PS, showed favorable gastrointestinal tolerability [13]. Moreover, since natural proteins are absorbed differently compared to free L-AAs in terms of speed, intestines exposed to free L-AAs find a non-physiological condition that may lead to the onset of oxidative stress and inflammation. Previous studies have focused on docosahexaenoic acid (DHA) [14] and glycomacropeptide (GMP)-based PS [15] on the evaluation of their effects in human intestinal Caco-2 cells. In the first study, DHA altered cytokine release and reduced H_2_O_2_-induced reactive oxygen species (ROS) and nitric oxide (NO) generation through the iNOS pathway; in the second study, the intestine oxidative and inflammatory status were considerably worsened by consuming L-AAs, whereas the redox state was positively restored and the inflammatory status was significantly reduced by GMP and the 1:1 free L-AA/GMP mixture, in the same cellular system. Given this, the purpose of this study is to investigate the impact of an SR-AAs PS available on the market for children with PKU on both the oxidative and inflammatory status in human intestinal Caco-2 cells, comparing its mechanism of action with that of a mixture of L-AAs. This cell line was selected as a widely accepted in vitro intestinal model for testing the efficacy and safety of natural and synthetic products, respectively [16]. Hence, we induced oxidative stress and inflammation at the cellular level using H_2_O_2_ and lipopolysaccharides (LPSs), respectively, and both L-AAs and SR-AAs were tested to evaluate if they were able to restore a more physiological condition. To reach this goal, experiments aimed at evaluating the direct antioxidant activity were performed using 2,2-diphenyl-1-picrylhydrazyl (DPPH) and Ferric Reducing Antioxidant Power (FRAP) assays, respectively. Then, the effects of both free AAs and SR-AAs on cellular viability were assessed through 3-(4,5-Dimethylthiazol-2-yl)-2,5-Diphenyltetrazolium Bromide (MTT) experiments. Finally, the ability of each tested sample to modulate the production of pro-inflammatory (IL-1β, IL-6, IFN-γ, and TNF-α) and anti-inflammatory (IL-10) cytokines in human intestinal Caco-2 cells, as well as lipid peroxidation, nitric oxide (NO), and ROS, was observed, offering new preclinical data that can clarify and support the efficacy of dietary therapy for the treatment and management of patients with PKU.

## 2. Materials and Methods

### 2.1. Chemicals

Various reagents and laboratory materials were sourced from different suppliers. Dulbecco’s Modified Eagle’s Medium (DMEM), L-glutamine, fetal bovine serum (FBS), phosphate-buffered saline (PBS), penicillin/streptomycin, chemiluminescent reagent, and 24- and 96-well plates were obtained from Euroclone (Milan, Italy). MTT [3-(4,5-dimethylthiazol-2-yl)-2,5-diphenyltetrazolium bromide], DPPH (1,1-diphenyl-2-picrylhydrazyl), TPTZ, Griess reagent, bovine serum albumin (BSA), RIPA buffer, the β-actin antibody, a fluorometric intracellular ROS kit, and an MDA assay kit were purchased from Sigma-Aldrich (St. Louis, MO, USA). Phenylmethyl-sulfonyl fluoride (PMSF), Na-orthovanadate inhibitors, and antibodies against rabbit Ig-horseradish peroxidase (HRP) and mouse Ig-HRP were supplied by Santa Cruz Bio-technology Inc. (Santa Cruz, CA, USA). The primary iNOS antibody was sourced from Cell Signaling Technology (Danvers, MA, USA), while the Complete Midi inhibitor cocktail was obtained from Roche (Basel, Switzerland). Mini Protean TGX pre-cast gel (7.5%) and Mini Nitrocellulose Transfer Packs were acquired from BioRad (Hercules, CA, USA). The qualitative and quantitative compositions of free AAs and SR-AAs are described in the following tables (Table 1 and Table 2).

#### Free AAs and SR-AAs Composition

SR-AAs are a mixture of amino acids free from phenylalanine and enriched with fats and carbohydrates, indicated for the dietary management of PKU from the third year of life. SR-AAs are available as tablets that can be taken with water or other permitted liquids. The qualitative and quantitative compositions of SR-AAs are described in Table 1. The free AAs used in this study are a phenylalanine-free drink mix, containing all the other AAs and carbohydrates. The contents of one sachet can be taken with a small amount of water.

### 2.2. DPPH (2,2-Diphenyl-1-Picrylhydrazyl Radical Scavenging) Assay

To evaluate antioxidant activity, a 2,2-diphenyl-1-picrylhydrazyl (DPPH) radical assay was performed following a slightly adapted version of the standard method [17]. For DPPH experiments, both free amino acid (AA) and slow-release amino acid (SR-AA) samples were fully dissolved in H_2_O to create stock solutions at a concentration of 100 mg/mL. In this procedure, different concentrations of free AAs and SR-AAs (ranging from 1.0 to 5.0 mg/mL) were combined with a DPPH solution (12.5 μM in methanol, 45 μL per well). The reaction, aimed at neutralizing DPPH radicals, was conducted at room temperature in the absence of light. After a 30 min incubation period, absorbance was recorded at 520 nm.

### 2.3. Assessment of Ferric Reducing Antioxidant Power (FRAP)

The FRAP assay was conducted to assess the ability of a sample to reduce ferric ions (Fe^3+^) to ferrous ions (Fe^2+^) [18]. For FRAP experiments, both free amino acid (AA) and slow-release amino acid (SR-AA) samples were completely dissolved in H_2_O to obtain stock solutions at a concentration of 100 mg/mL. To obtain final concentrations of 0.1, 1.0, and 5.0 mg/mL, a mixture of 140 µL of FRAP reagent and 10 µL (15×) of free AAs and SR-AAs was prepared. The FRAP reagent itself was made by combining 1.3 mL of a 10 mM TPTZ solution (Sigma-Aldrich, Milan, Italy) dissolved in 40 mM HCl, 1.3 mL of 20 mM FeCl_3_ × 6 H_2_O, and 13 mL of 0.3 M acetate buffer (pH 3.6). Following an incubation period of 30 min at 37 °C, the absorbance was recorded at 595 nm using a Synergy^TM^ HT multimode microplate reader.

### 2.4. Cell Culture

Caco-2 cells, originally obtained from INSERM (Paris, France), were routinely subcultured at 50% confluence and maintained at 37 °C in an environment composed of 90% air and 5% CO_2_ [19]. The cells were cultured in Dulbecco’s Modified Eagle’s Medium (DMEM) with an addition of 10% heat-inactivated fetal bovine serum (FBS; Hyclone Laboratories, Logan, UT, USA), 25 mM glucose, 3.7 g/L sodium bicarbonate (NaHCO_3_), 4 mM stable L-glutamine, and 1% non-essential amino acids, as well as 100 U/L penicillin and 100 μg/L streptomycin, to create a complete growth medium.

### 2.5. 3-(4,5-Dimethylthiazol-2-Yl)-2,5-Diphenyltetrazolium Bromide (MTT) Assay

Caco-2 cells were seeded at a density of 30,000 cells per well in 96-well plates and subsequently exposed to free AAs and SR-AAs at concentrations ranging from 0.1 to 100.0 mg/mL, or to the vehicle control, in complete growth medium. The treatment was maintained for 48 h at 37 °C in a 5% CO_2_ atmosphere. After removing the treatment solutions, a filtered solution of 3-(4,5-dimethylthiazol-2-yl)-2,5-diphenyltetrazolium bromide (MTT) (100 µL per well) was added. Following a two-hour incubation at 37 °C with 5% CO_2_, the MTT solution was discarded, and 100 µL of lysis buffer (8 mM HCl + 0.5% NP-40 in DMSO) was introduced into each well. The absorbance at 575 nm was recorded using a Synergy H1 fluorescence plate reader (Biotek, Bad Friedrichshall, Germany) after ten minutes of gentle shaking [20].

### 2.6. Fluorometric Intracellular ROS Assay

To carry out the experiments, Caco-2 cells were plated at a density of 30,000 cells per well in a black 96-well plate and incubated overnight in growth medium. The next day, the medium was removed, and 50 μL per well of complete DMEM along with 50 μL per well of the Master Reaction Mix were added. The cells were then incubated at 37 °C with 5% CO_2_ for one hour, ensuring protection from light exposure. Subsequently, 10 μL of 11× free AAs and SR-AAs were introduced to the wells to reach final concentrations of 1.0 and 5.0 mg/mL, followed by 24 h of incubation in the dark at 37 °C. To induce oxidative stress and generate reactive oxygen species (ROS), the cells were treated with 10 μL of H_2_O_2_ at a final concentration of 1.0 mM and incubated in the dark at 37 °C for one hour. Fluorescence signals (ex./em. 490/525 nm) were then measured using a Synergy H1 microplate reader (Biotek, Bad Friedrichshall, Germany) [21,22].

### 2.7. Evaluation of Lipid Peroxidation Through Malondialdehyde (MDA) Assay

Caco-2 cells were seeded at a density of 250,000 cells per well in a 24-well plate. The following day, the cells were treated with free AAs and SR-AAs at final concentrations of 1.0 and 5.0 mg/mL, maintaining incubation at 37 °C with 5% CO_2_ for 24 h. After the incubation period, oxidative stress was induced by exposing the cells to either 1 mM H_2_O_2_ or a vehicle control for 60 min. The cells were then collected and homogenized in 150 µL of ice-cold MDA lysis solution containing 1.5 µL of BHT (100×). After centrifugation at 13,000× *g* for 10 min, the insoluble fraction was removed by filtering through a 0.2 µm membrane. To facilitate the formation of the MDA-TBA adduct, 300 µL of TBA solution was added to each sample and incubated at 95 °C for 60 min, followed by a cooling step in an ice bath for 10 min to bring the samples to room temperature. For MDA detection, 100 µL of each reaction mixture was transferred to a 96-well plate, and absorbance at 532 nm was measured using a Biotek Synergy H1 fluorescent plate reader [23].

### 2.8. Nitric Oxide Level Evaluation on Caco-2 Cell

Caco-2 cells were plated at a density of 150,000 cells per well in a 24-well plate. The following day, they were exposed to free AAs and SR-AAs for 24 h to achieve a final concentration of 1.0 mg/mL and maintained at 37 °C in a 5% CO_2_ incubator. After treatment, oxidative stress and inflammation were induced using H_2_O_2_ (final concentration: 1.0 mM) and LPS (final concentration: 1 μg/mL), respectively [13,14]. The level of nitric oxide (NO) was assessed using the Griess assay [15]. In brief, 1.0 g of Griess reagent powder was dissolved in 25.0 mL of distilled water, and 50.0 μL of this solution was mixed with an equal volume (50.0 μL) of culture supernatant. The mixture was incubated in the dark at room temperature for 15 min. Absorbance was then recorded at 540 nm using a Biotek Synergy H1 fluorescent plate reader [24,25].

### 2.9. Western Blot Analysis

Caco-2 cells (150,000 per well) were seeded in a 24-well plate and treated with 1.0 mg/mL of free AAs and 1.0 mg/mL of SR-AAs for 24 h. Following incubation, oxidative and inflammatory stimuli were induced using H_2_O_2_ (1.0 mM) and LPS (1 μg/mL), respectively, or a vehicle control for an additional 24 h. The cell culture media were then collected in ice-cold microcentrifuge tubes for subsequent Griess assay and cytokine quantification. Meanwhile, the cells were scraped into 30 µL of ice-cold lysis buffer [RIPA buffer supplemented with an inhibitor cocktail, 1:100 PMSF, and 1:100 Na-orthovanadate] and transferred to an ice-cold microcentrifuge tube. The lysates were centrifuged at 15,000× *g* for 15 min at 4 °C, and the supernatant was carefully collected into a new ice-cold tube. Protein concentration was determined using the Bradford assay, and 50 μg of total protein was loaded onto a pre-cast 7.5% SDS-PAGE gel, followed by electrophoresis at 130 V for 45 min. The gel was then pre-equilibrated in 0.04% SDS in water for 15 min at room temperature before being transferred onto a nitrocellulose membrane using a Trans-Blot Turbo system (1.3 A, 25 V, 7 min). After blocking with either milk or BSA, the membrane was probed with primary antibodies targeting iNOS and β-actin. HRP-conjugated secondary antibodies and a chemiluminescent reagent were used for visualization, and protein signals were quantified using Image Lab Software version 5.1 (Bio-Rad, Hercules, CA, USA). β-actin served as an internal control to account for loading differences.

### 2.10. Pro-Inflammatory and Anti-Inflammatory Secreted Cytokines Quantification

Cytokine levels were measured using Human Quantikine^®^ ELISA kits (R&D Systems, Minneapolis, MN, USA) following the manufacturer’s guidelines. In brief, supernatants from treated and LPS-stimulated Caco-2 cells were centrifuged at 15,000× *g* for 10 min at 4 °C, after which the pellet and insoluble material were discarded. For the assay, 100 µL of each sample was added to the designated wells, and the microplate was incubated at room temperature (RT) for 2 h. Following incubation, the wells were emptied, washed four times with 300 µL of Wash Buffer solution, and then 200 µL of Conjugate solution was added to each well. The plate was incubated again at RT for 2 h. After incubation, the wells were washed four times with 300 µL of Wash Buffer solution before adding 200 µL of Substrate Solution. The plate was then incubated in the dark at RT for 1 to 4 h. Finally, 50 µL of Stop Solution was added to halt the reaction, and absorbance was measured at 450 nm and 540 nm using a Synergy H1 plate reader (BioTek Instruments, Winooski, VT, USA).

### 2.11. Statistical Analysis

All data were presented as mean ± standard deviation (s.d.), with statistical significance set at *p*-values < 0.05. Normality of the data was assessed using the D’Agostino and Pearson test. Since all datasets followed a normal distribution (*p*-values < 0.05), statistical comparisons were conducted using one-way ANOVA, followed by Tukey’s post hoc test (GraphPad Prism 10, GraphPad Software, La Jolla, CA, USA).

## 3. Results

### 3.1. FRAP and DPPH Assays Are Used to Evaluate the Free AAs’ and SR-AAs’ Capacity to Scavenge Radicals In Vitro

**Figure 1:**
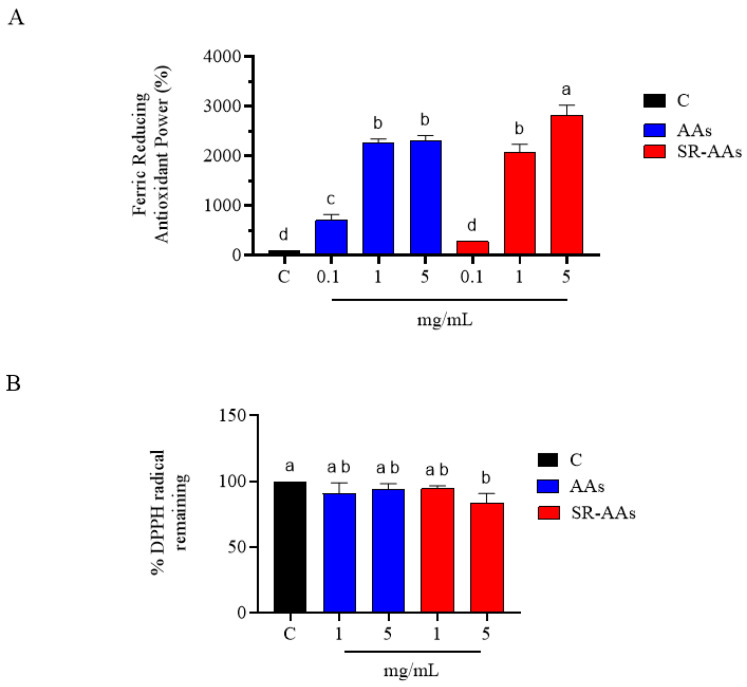
The FRAP (**A**) and DPPH (**B**) tests were used to measure the in vitro radical scavenging activity of free AAs and SR-AAs, respectively. The data are the mean ± standard deviation of four triplicate determinations. Different lowercase letters indicate a significant difference (*p* < 0.05) between different treatments. C: control.

The antioxidant properties of free AAs and SR-AAs were assessed through the FRAP and DPPH assays. Figure 1A shows that free AAs augmented the FRAP in a dose-dependent manner, namely by 687.0 ± 120.0%, 2300 ± 320.0% and 2370 ± 290.0% at 0.1, 1.0 and 5 mg/mL, respectively, whereas SR-AAs augmented the FRAP by 277.3 ± 9.09%, 2073 ± 163.3%, and 2823 ± 201.6% at the same concentrations (Figure 1A), showing a dose–response trend. In addition, Figure 1B shows that free AAs scavenged the DPPH radical by 9.3 ± 8.2% and 5.0 ± 3.8% at 1.0 and 5.0 mg/mL, respectively, while the SR-AA sample reduced the DPPH radical by 5.69 ± 2.39% and 16.57 ± 7.34% at 1.0 and 5.0 mg/mL, respectively (Figure 1B). These results confirm the free AA activity demonstrated in our previous research [15], while giving new information about the SR-AAs’ formulation, whose better antioxidant activity, particularly visible at 5 mg/mL, could be attributed to the formulation’s ability to improve the amino acids’ stabilization, preserving their antioxidant potential.

### 3.2. Assessment of the Impact of Free AAs and SR-AAs on the Viability of Caco-2 Cells

**Figure 2:**
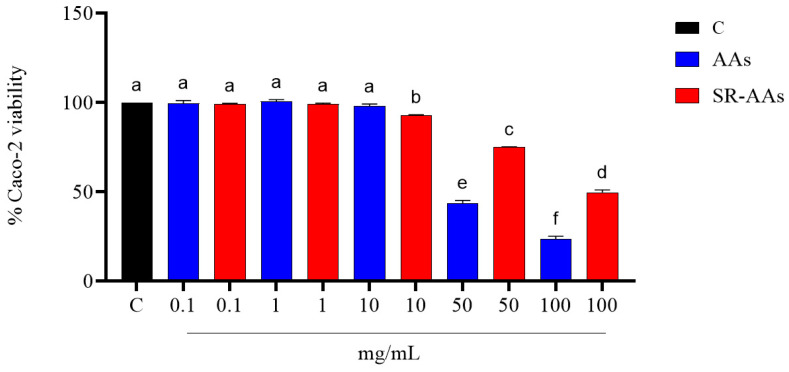
Effects of free AAs and SR-AAs on human intestine Caco-2 cells. The data points show the mean ± standard deviation (s.d.) derived from four independent experiments performed in duplicate. Statistical analysis was conducted using one-way ANOVA followed by Tukey’s post hoc test. Different lowercase letters indicate a significant difference (*p* < 0.05) between different treatments. C: control sample.

Based on our previous results, cellular evaluations of free AAs’ and SR-AAs’ antioxidant properties were carried out. Prior to experiments on Caco-2 cells, MTT tests were conducted to make sure the compounds did not affect the vitality of the cells. The SR-AA sample is a safer product than the free AA sample, according to the results. In fact, free AAs reduced the cellular viability by 54.0 ± 4.1% and 75.1 ± 2.6% at 50.0 and 100.0 mg/mL, respectively, compared with SR-AAs, which decreased the Caco-2 cells’ viability by 23.4 ± 0.3% and 50.5 ± 2.1% at the same concentrations (Figure 2).

### 3.3. Free AAs and SR-AAs Exert Antioxidant Activity in Human Intestinal Caco-2 Cells

**Figure 3:**
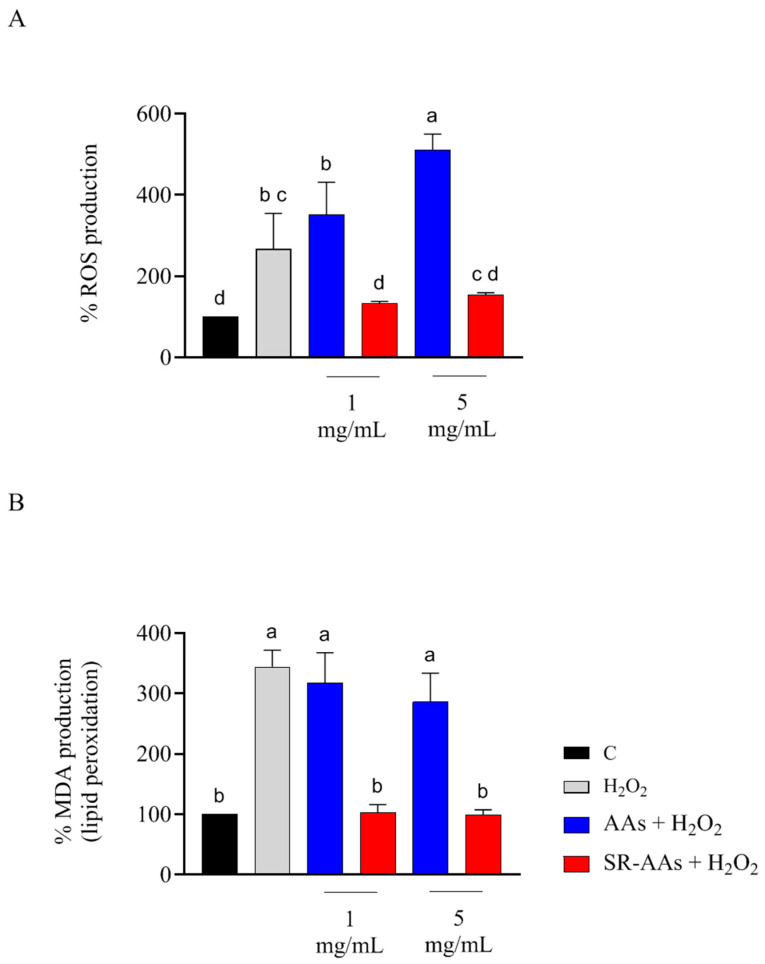
Impact of free AAs and SR-AAs on the regulation of ROS (**A**) and MDA (**B**) produced by H_2_O_2_ in human intestinal Caco-2 cells. The data are expressed as the mean ± s.d. from six measurements conducted in triplicate. Different lowercase letters indicate a significant difference (*p* < 0.05) between different treatments. C: control.

The ability of free AAs and SR-AAs to regulate ROS overproduction caused by H_2_O_2_ was assessed in cellular experiments. H_2_O_2_ (1 mM) was used to induce oxidative stress in human intestinal Caco-2 cells without compromising cell viability. The results demonstrated that Caco-2 cells exposed to H_2_O_2_ alone showed a significant increase in ROS levels, reaching 266.9 ± 87.82% compared to control cells. As illustrated in Figure 3A, free AAs further elevated intracellular ROS levels induced by H_2_O_2_, reaching 352.0 ± 79.32% and 511.0 ± 38.5% at concentrations of 1.0 mg/mL and 5.0 mg/mL, respectively. In contrast, SR-AAs reduced ROS levels to 133.3 ± 4.3% and 153.5 ± 5.9%, respectively, at the same concentrations (Figure 3A). Furthermore, MDA levels were measured to assess the effects of free AAs and SR-AAs on H_2_O_2_-induced lipid peroxidation in Caco-2 cells. Consistent with the increase in ROS following treatment, intracellular lipid peroxidation rose sharply to 344.6 ± 27.66% compared to control cells. Pre-treatment with free AAs modulated MDA levels to 318.2 ± 49.8% and 286.3 ± 47.5% at 1.0 mg/mL and 5.0 mg/mL, respectively. Conversely, pre-treatment with SR-AAs significantly reduced MDA levels to 102.7 ± 13.5% and 99.2 ± 8.2% at the same concentrations (Figure 3B). Therefore, free AAs confirm that they are not able to exert any protective effect from free radicals, contrary to what was observed for SR-AAs where a dose-dependent antioxidant capacity is noted.

### 3.4. Free AAs and SR-AAs Modulate the Generation of NO and iNOS Expression in Intestinal Caco-2 Cells

**Figure 4:**
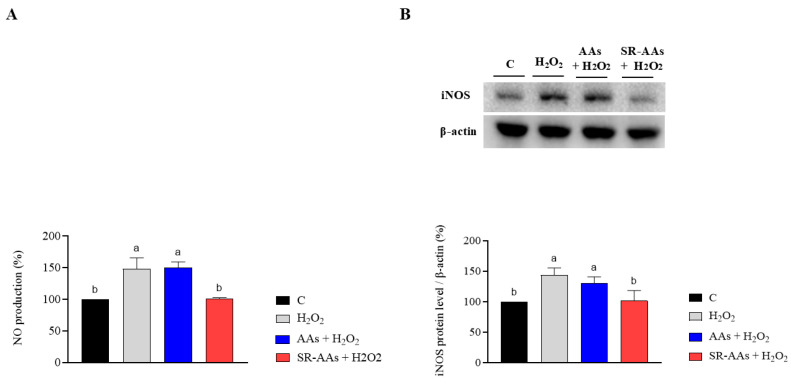
Effects of free AAs and SR-AAs on the levels of H_2_O_2_-induced NO generation (**A**) and iNOS protein production (**B**) in human intestinal Caco-2 cells, respectively. Data represent the mean ± s.d. of six measurements conducted in three replicates and in duplicate for immunoblotting analysis. Different lowercase letters indicate a significant difference (*p* < 0.05) between different treatments. C: control.

The impact of free AAs and SR-AAs on NO production following oxidative stress was assessed in human intestinal Caco-2 cells. Remarkably, treatment with H_2_O_2_ (1 mM) triggered oxidative stress, resulting in an increase in intracellular NO levels to 148.8 ± 16.9% (Figure 4). Figure 4A shows that the H_2_O_2_-induced NO overproduction was enhanced to 149.8 ± 9.5% at 1.0 mg/mL by pre-treatment with free AAs. In contrast, pre-treatment with SR-AAs reduced the NO production by up to 100.8 ± 1.9% tested at 1.0 mg/mL (Figure 4A). Similarly, after oxidative stress induction, the effects of free AAs and SR-AAs on iNOS protein levels were evaluated using Western blot tests, which identified and quantified the iNOS protein band at 130 kDa. The results (Figure 4B) showed that the iNOS protein rose to 143.9 ± 11.6% following H_2_O_2_ treatment (1 mM). Pre-treatment with free AAs modulates H_2_O_2_-induced iNOS levels, increasing them to 130.8 ± 10.0% at a concentration of 1.0 mg/mL compared to control cells, while, the SR-AA sample lowered the H_2_O_2_-induced iNOS protein level up to 101.1 ± 17.3% at 1.0 mg/mL (Figure 4B).

**Figure 5:**
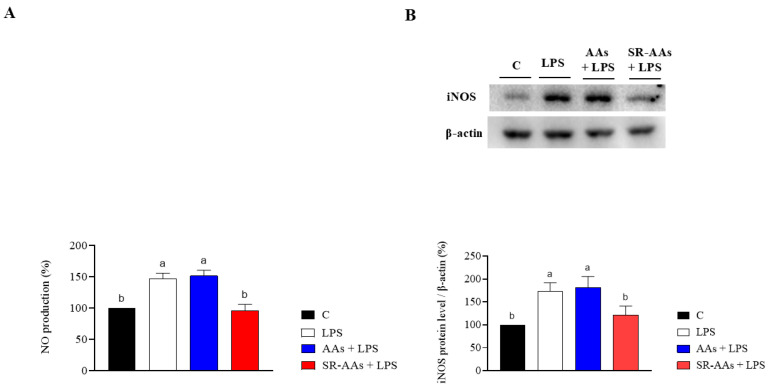
Evaluation of the effects of free AAs and SR-AAs on LPS-induced NO production (**A**) and iNOS protein levels (**B**) in human intestinal Caco-2 cells. Data represent the mean ± s.d. of six measurements performed in triplicate and duplicate for immunoblotting analysis. Different lowercase letters indicate a significant difference (*p* < 0.05) between different treatments. C: control.

These outcomes show that, in intestinal Caco-2 cells, exposure to LPS caused an inflammatory state that resulted in increased NO levels of 147.5 ± 8.3% (Figure 5A) and iNOS level productions of 174.2 ± 17.7% (Figure 5B). The administration of free amino acids led to an increase in NO production, reaching 151.3 ± 9.3% (Figure 5A), and iNOS production up to 181.3 ± 24.6% (Figure 5B) at 1.0 mg/mL. Otherwise, pre-treatment SR-AAs reduced the NO production until 95.7 ± 10.1% (Figure 5A) and the iNOS production up to 121.0 ± 20.4% (Figure 5B) at 1.0 mg/mL.

### 3.5. Free AAs and SR-AAs Exert Anti-Inflammatory Activity in Human Intestinal Cells

**Figure 6:**
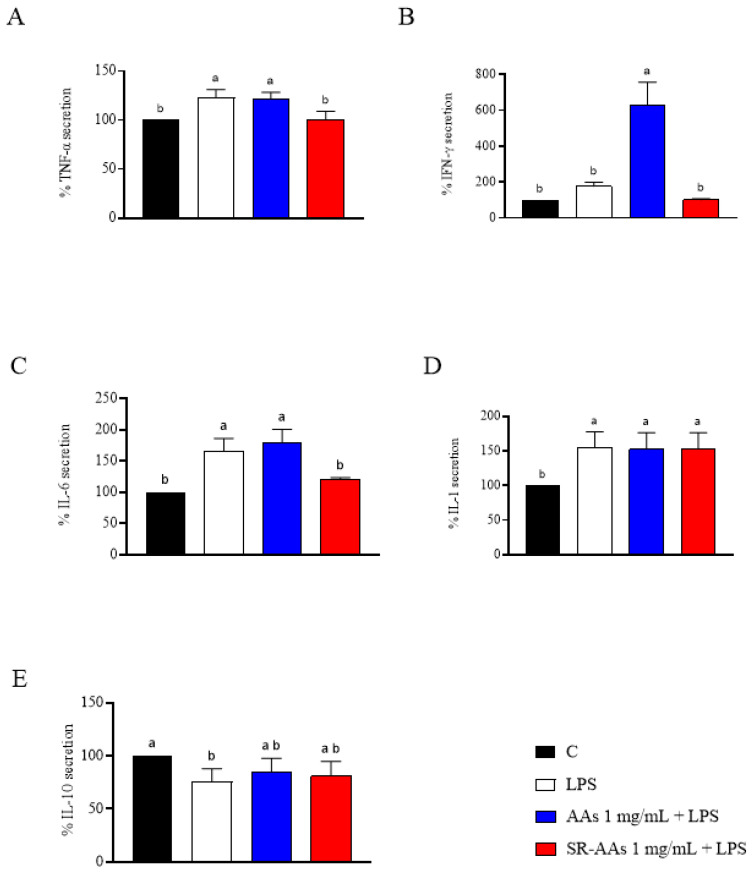
Assessment of the effects of free AAs and SR-AAs on the production of pro-inflammatory cytokines TNF-α (**A**), IFN-γ (**B**), IL-6 (**C**), and IL-1β (**D**) and the anti-inflammatory cytokine IL-10 (**E**) in Caco-2 cells induced by lipopolysaccharide (LPS). Data represent the mean ± s.d. of six measurements performed in triplicate. All datasets were analyzed using one-way ANOVA followed by Tukey’s post hoc test. Different lowercase letters indicate a significant difference (*p* < 0.05) between different treatmentss. C: control.

As expected, LPS stimulation of Caco-2 cells led to an increase in pro-inflammatory cytokines (TNF-α, IFN-γ, IL-6, and IL-1β) and a reduction in the anti-inflammatory cytokine IL-10 (Figure 6). Specifically, LPS stimulation resulted in the following changes in cytokine levels: TNF-α was augmented by 123.0 ± 8.2%; IFN-γ by 177.1 ± 20.9%; IL-6 by 166.4 ± 19.7%; and IL-1β by 155.3 ± 22.3%. IL-10 decreased by 23.1 ± 10.9%. Furthermore, in the presence of free AAs, the levels of pro-inflammatory cytokines were not reduced. In fact, the TNF-α increased to 121.0 ± 7.3%; IFN-γ was augmented by 632.5 ± 124.2%, IL-6 by 180.2 ± 20.3%, and IL-1β increased by 152.4 ± 24.1%, while the anti-inflammatory cytokine IL-10 remained unchanged, showing no significant modulation. Conversely, results in Figure 6 indicate that pre-treatment with SR-AAs significantly reduced the pro-inflammatory cytokines. Specifically, TNF-α was decreased to 100.7 ± 8.1%, IFN-γ was reduced to 100.0 ± 6.7% and IL-6 was decreased to 121.0 ± 2.4%. However, IL-1β levels showed a slight increase to 153.7 ± 22.8%, and the anti-inflammatory cytokine IL-10 was reduced to 81.0 ± 13.7%.

## 4. Discussion

Scientific studies suggest that the speed of protein digestion and amino acids absorption has an important effect on protein metabolism because of the postprandial protein catabolism [10,26]. In the present research, we worked with a commercially available slow-release amino acids PS. The slow-release formula has characteristics that make it different from the traditional blend of L-AAs; in particular, in the prolonged-release formula the amino acids are encapsulated in a cellulose layer, which allows for protection from the acid pH which is physiologically present in the stomach. In detail, this is possible thanks to a particular technological system based on sodium alginate: sodium alginate, in the acidic environment of the stomach, is transformed into insoluble alginic acid and forms a clot. The release of amino acids is hindered and therefore slowed down. By slowly releasing the amino acids, the micro tablets reproduce the same advantages as slow-digested proteins, resulting in a significant improvement in protein status. In the case of the consumption of the traditional free L-AAs, the amino acids rapidly reach the intestine, where they are immediately absorbed and transported to the blood¸ thus promoting the protein catabolism (https://www.piamfarmaceutici.com/sostituti-proteici-a-lento-rilascio, accessed on 9 December 2024). Given the similar nutritional compositions in terms of amino acids, we can assume that the observed results are primarily attributable to the release modality.

The results from our study clearly reveal that the treatment with free AAs significantly worsens the intestinal oxidative and inflammatory status, confirming the effects already seen [15], and that the SR-AAs positively restore the physiological oxidative and inflammatory status. Thus, SR-AA formulation helped to significantly counteract the pro-oxidate and inflamed cellular phenotypes. By providing a controlled supply of amino acids, slow-release formulation prevents the sudden spikes in AA levels that can trigger oxidative stress and inflammation. Additionally, SR-AAs can contribute to maintaining proper cellular function and integrity, reducing the likelihood of damage and impairment. Evidence from our study demonstrates that free AAs are unable to scavenge the DPPH radicals, whereas SR-AAs can slightly reduce them. Moreover, both samples display FRAP activity. According to this and considering that the intestine serves as a barrier to these foods, an in-depth investigation at the intestinal cell level was conducted. Human intestinal Caco-2 cells have been utilized as an intestinal model [16] and initial MTT assays indicated that treatment with free AAs (50.0 mg/mL) decreased Caco-2 cell viability in comparison to the control, while the Caco-2 cells viability was less decreased by the SR-AAs, at the same concentrations (Figure 2). This suggests that the slow-release sample halves cell mortality compared to free AAs, supporting the product’s safety. Moreover, in contrast to free AAs, SR-AAs restore the physiological intracellular ROS levels in Caco-2 cells at 1.0 and 5.0 mg/mL following H_2_O_2_ stimulation. Specifically, free AAs significantly increased intracellular ROS induced by H_2_O_2_, while SR-AAs notably reduced their pro-oxidant effects, demonstrating antioxidant activity at 1.0 and 5.0 mg/mL (Figure 3A). Consistent with the modulation of ROS, free AAs were ineffective in reversing H_2_O_2_-induced lipid peroxidation in Caco-2 cells, whereas SR-AAs, at the same concentrations, effectively reduced H_2_O_2_-induced lipid peroxidation to baseline levels, neutralizing the pro-oxidant effects on lipid peroxidation (Figure 3B). These findings are consistent with clinical data showing that free AAs promote nitrogen excretion and amino acid oxidation, reducing the availability of these nutrients for cellular functions [27]. This altered nitrogen balance, observed in patients with chronic altered metabolic conditions, is linked to mitochondrial β-oxidation [27]. This process is characterized by a reduction in the ATP/available oxygen ratio that forces the cell to use large quantities of essential amino acids as intermediates in the Krebs cycle, and it is responsible of ROS generation [28,29]. Furthermore, our findings showed that SR-AAs had a direct impact on the levels of iNOS protein in CaCo-2 cells, which allowed them to restore the normal levels of NO production generated by H_2_O_2_ and LPS, in contrast to the free L-AA sample (Figure 4 and Figure 5). These findings strongly indicate that NO is a crucial signaling molecule involved in linking oxidative stress and inflammation. Therefore, our results show that SR-AAs have a significant anti-inflammatory effect by reducing the secretion of LPS-induced pro-inflammatory cytokines (IFN-γ, TNF-α, and IL-6). Conversely, the free AA sample has pro-inflammatory properties since it substantially increases LPS-induced IFN-γ and IL-6 secretion (Figure 6).

Very similar behavior has been seen in a previous study that investigated the effects of GMP on intestinal cells. GMP-based PSs are helpful products for the dietary management of PKU patients because they can improve dietary adherence due to better palatability, as well as enhance body composition and gut microbiota status in children with PKU. The study showed that GMP could reduce oxidative stress and inflammation in Caco-2 cells; in contrast, free L-AAs have been shown to exert no antioxidant or anti-inflammatory effects on the same cell line [15].

Several studies have reported the presence of inflammation and oxidative stress in PKU, which may be linked either to the pathology itself or to dietary interventions [30,31].

Despite existing clinical studies highlighting the association between PKU and oxidative stress, there remains a gap in the literature regarding the specific impact of slow-release mixtures on inflammatory status and oxidative stress in PKU patients. To date, no studies have assessed the role of slow-release mixtures in this context, leaving a lack of data on their systemic-level effects in PKU management.

From a pathophysiological perspective, this inflammatory state can contribute to the development of comorbidities [31]. Evidence suggests that the DNA damage in PKU patients is associated with phenylalanine blood levels, with oxidative DNA damage increasing in individuals with higher blood Phe levels and poor dietary compliance [32]. In our study, regarding inflammation, we observed that the SR-AAs reduced pro-inflammatory cytokines, particularly TNF-α, IFN-γ, and IL-6. Specifically, they have been associated with systemic conditions such as insulin resistance and cardiovascular disease, immune dysregulation and neuroinflammation, and metabolic disorders, chronic inflammation, and neurodegeneration [33,34,35]. Therefore, further investigating the role of these cytokines in patients could provide valuable insights into their contribution to comorbidities associated with the disease. Understanding their systemic impact may help refine therapeutic strategies, especially considering the aging process in PKU patients, where chronic inflammation and metabolic disturbances could exacerbate long-term health outcomes [36].

The purpose of slow-release formulations is to allow a physiological absorption of amino acids that could reduce gut inflammatory state, and, furthermore, ameliorate the composition of the microbiota. A recent systematic review and meta-analysis on gut microbiota in patients with PKU [37] identified significant reductions in several taxonomic groups in individuals with PKU compared to the control group, also revealing the potential dietary influence in adult PKU populations. In fact, children with PKU have shown to have deficiency of some beneficial microbes, especially *Fecalobacterium prausnitzii*, important for gut health [38]. Moreover, children with PKU show a high prevalence of *Blautia* genera, which is related to pro-inflammatory effects [37]. Considering that nutritional intervention is the main treatment for subjects with hyperphenylalaninemia, and the disruption of the pro-oxidant/antioxidant balance in PKU patients is strongly correlated to diet, it appears essential to find Phe-free PS with a nutritional composition that can mitigate the negative effects while promoting benefits instead [39]. As oxidative stress is also involved in the development of cardiovascular diseases (CVDs), scientific studies have explored the relationship between cardiovascular risk factors and obesity, metabolic profile, and dietary glycemic index (GI) [40], showing that pediatric patients with classical PKU are at risk for the development of CVDs, regardless of obesity and GI, and children adhering to the diet show no different cardiovascular risks than the healthy population [41].

Together with the results from the current study, this clearly highlights the need for well-researched PS, both in vivo and in vitro, to assess not only patients’ preference and usage, along with metabolic control of the disease, but also the impact on inflammation, which is closely linked to the risk of developing chronic diseases.

Moreover, the role of gut microbiota is increasingly recognized as central to systemic health. In patients, such as patients with PKU, who must adhere to a restrictive lifelong diet, it becomes even more crucial to provide bioactive components that can have effects at a systemic level. However, long-term studies are necessary to evaluate potential changes in efficacy, particularly considering the individual variability among PKU patients.

## 5. Conclusions

In conclusion, our findings provide new preclinical insights into the mechanisms of action of free amino acids and slow-release AAs on intestinal cells. This supports SR-AAs as a valid and health-promoting option for the dietary management of individuals with Phenylketonuria (PKU), increasing attention to nutrition, not only for managing the metabolic condition itself but also for ensuring that nutritional needs common to all children are met. This approach aims to promote overall health status and reduce the risk of developing chronic diseases from adolescence onward. However, it is important to recognize a few limitations. Notably, the absence of simulated gastrointestinal digestion in the study design limits the ability to assess the full physiological relevance of the SR-AAs formulation. Indeed, digestion and absorption dynamics are crucial factors in determining how amino acids are processed in vivo and their impact on systemic effects. Furthermore, although intestinal cells were used as an in vitro model in this study, they do not fully represent the complexity of the human gastrointestinal system, including microbiota interactions and the process of absorption over time. Therefore, incorporating simulated digestion systems could help validate these promising results in a more physiologically accurate context, providing a deeper understanding of how the SR-AAs behave in the human digestive system.

## Figures and Tables

**Table 1:** Nutritional composition of the SR-AAs product used in the experiments. This product can be used from 3 years of age.

Nutritional Values	Mean Values per 100 g
Energy (Kj)	1678
Energy (Kcal)	396
Total Fats (g)	3.6
Saturated Fats (g)	3.59
Carbohydrates (g)	13
Sugars (g)	0
Total Fiber (g)	3.7
Total Protein Equivalents (g)	70.7
L-Alanine (g)	3.07
L-Arginine (g)	4.90
L-Aspartic-Acid (g)	7.76
L-Cystine (g)	2.01
Glycine (g)	7.68
L-Glutamine (g)	6.02
L-Histidine (g)	3.07
L-Isoleucine (g)	5.31
L-Leucine (g)	8.27
L-Lysine (g)	5.50
L-Methionine (g)	1.42
L-Phenylalanine (g)	0
L-Proline (g)	5.54
L-Serine (g)	3.42
L-Threonine (g)	5.31
L-Tryptophan (g)	1.65
L-Tyrosine (g)	7.78
L-Valine (g)	6.13
L-Carnitine (g)	0.08
L-Taurine (g)	0.12
Salt (g)	1

**Table 2:** Nutritional composition of the free AAs mixture used in the experiments.

Nutritional Values	Mean Values per 100 g
Energy (Kj)	1456
Energy (Kcal)	343
Total Fats (g)	/
Saturated Fats (g)	/
Carbohydrates (g)	44
Sugars (g)	40.6
Toral Fiber (g)	/
Total Proteins Equivalents (g)	41.65
L-Alanine (g)	2.05
L-Arginine (g)	3.6
L-Aspartic-Acid (g)	3.4
L-Cystine (g)	1.35
Glycine (g)	3.2
L-Glutamine (g)	2.5
L-Histidine (g)	2.05
L-Isoleucine (g)	3.2
L-Leucine (g)	5.45
L-Lysine (g)	3.7
L-Methionine (g)	0.85
L-Phenylalanine (g)	0
L-Proline (g)	3.85
L-Serine (g)	2.35
L-Threonine (g)	2.7
L-Tryptophan (g)	1.1
L-Tyrosine (g)	4.85
L-Valine (g)	3.5
L-Carnitine (g)	0.04
Taurine (g)	0.07
Salt (g)	<0.01
Sodium (mg)	<5
Potassium (mg)	35

## Data Availability

Data are contained within the article.
